# Biomarkers of Exposure to Zearalenone in In Vivo and In Vitro Studies

**DOI:** 10.3390/toxins14050291

**Published:** 2022-04-20

**Authors:** Paula Llorens, Marta Herrera, Ana Juan-García, Juan José Payá, Juan Carlos Moltó, Agustín Ariño, Cristina Juan

**Affiliations:** 1 Laboratory of Food Chemistry and Toxicology, Faculty of Pharmacy, University of Valencia, 46100 Valencia, Spain; paullo3@uv.es (P.L.); ana.juan@uv.es (A.J.-G.); juanpan2@alumni.uv.es (J.J.P.); j.c.molto@uv.es (J.C.M.); cristina.juan@uv.es (C.J.); 2 Instituto Agroalimentario de Aragón—IA2 (Universidad de Zaragoza-CITA), Facultad de Veterinaria, 50013 Zaragoza, Spain; aarino@unizar.es

**Keywords:** mycotoxins, zearalenone, biomarkers, α-zearalenol, β-zearalenol

## Abstract

The measurement of human exposure to mycotoxins is necessary for its association with adverse health effects. This exposure is usually estimated from contamination levels of foodstuffs, which are the primary source of toxin exposure, and data on food consumption patterns. However, variations in contamination level, intestinal absorption, toxin distribution, and excretion lead to individual variations in toxin exposure that can be more readily measured with a biomarker. This review deals with the latest literature information about ZEN biomarkers in humans, animals, and cell line cultures. Their presence in urine, biomarkers that have effects in the kidney, liver, reproductive system and blood and biomarkers of cell response have been reported. It has highlighted the importance of determining α-zearalenol and β-zearalenol biomarkers to estimate the probable dietary intake (PDI) of a specific population or to characterize the severity of exposure to ZEN in animals or cell lines. α-ZEL and β-ZEL are cytotoxic by inhibiting cell proliferation, total protein and DNA syntheses, in this sense, an induction of expression proteins Hsp27 and Hsp70 was observed, and an increase in gene expression (TLR4, NF-kBp65, TNF-α, IL-1β, IL-6, IL-8, MGMT, α-GST, Hsp70, Nrf2, L-Fabp, HO-1, MAPK8), the determination of which indicates an oxidative stress effect. The integrity of the cell or tissue membrane is assessed by lactate dehydrogenase (LDH), which increase at exposure of ZEN (84.2 µM), and the proportions of some fatty acids of the renal tissue membrane were increased at treatments with ZEN. This review allows starting future studies of animal and population exposure in parallel with those of health effects works.

## 1. Introduction

Zearalenone (ZEN) is a mycotoxin with a non-steroidal structure and estrogenic activity due to it mimicking 17-β-estradiol [1]. ZEN can bind competitively to estrogenic receptors in various animal species, and it has been recognized as a mammalian endocrine disruptor in some animal species in both males and females [2,3].

ZEN is produced mainly by fungi *Fusarium graminearum* and *F. culmorum*, among others, such as *F. equiseti* or *F. verticillioides*. These soil-borne fungi are common in warm and temperate climates, causing illness in hosts that ingest significant quantities of ZEN, which can be found in cereal crops, mainly corn, wheat, rice, barley, sorghum, rye, and in many cereal-based foods such as breakfast cereals, rolls, pasta, snacks and vegetable oils [4].

According to the Panel of Contaminants in the Food Chain (CONTAM), the presence of ZEN in corn (33%) and its average level (15 µg/kg) is significantly higher than in other cereal and food products in Europe [5]. ZEN contamination not only affects unprocessed cereals, but also milling fractions, germ and bran, with high mean levels up to 33 µg/kg in wheat bran. In the case of vegetable oils, 86% of samples were positive for ZEN with a mean level of 72 µg/kg. In general, the raw cereal cleaning by-products (powder and hulls, among others) were characterized by 3- to 30-fold higher zearalenone concentrations than the cleaned cereal grains, while bran contained up to 2-fold higher concentrations [5,6].

In 2011, the European Food Safety Authority (EFSA) conducted a detailed report based on the mentioned ZEN occurrence about the major food categories and food consumption data for different consumer groups. Hence, a tolerable daily intake (TDI) of 0.25 µg/kg body weight/day was established, expressed as ZEN equivalents for ZEN and its modified forms (phase I and phase II metabolites) based on the results observed in the animal species most sensitive to ZEN [5,7].

After oral ingestion, ZEN is metabolized by the liver and intestine. Despite its high bioavailability, it is difficult to determine its absorption levels given the enterohepatic circulation [8]. The International Agency for Research on Cancer (IARC) evaluated ZEN and it was not classified as human carcinogens (Group 3) [9].

**Figure 1:**
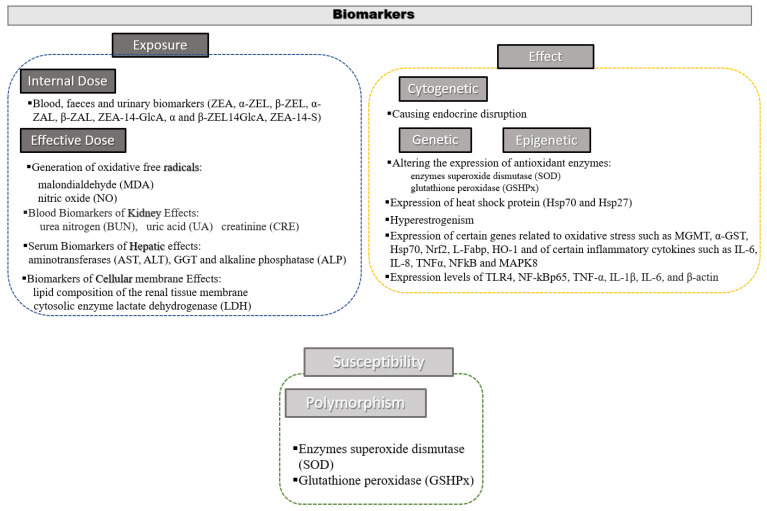
Biomarkers of exposure, effect and susceptibility of zearalenone.

Biomarkers are used to quantify the exposure to certain toxins and measure the severity of toxicity [10]. Then, a mycotoxin biomarker is a product from the biochemical metabolism pathway, specific, sensitive, quantifiable, and easily measurable, at adequate concentrations. A biomarker allows for observing adverse effects on human or animal health due to mycotoxin exposure. Thus, understanding the biotransformation of mycotoxins and the identification of reliable biomarkers is important for accurate risk assessment of mycotoxin exposure. Biomarkers are classified as indicators of exposure, effect, or susceptibility [11]. It can be considered a biomarker, the original substance itself, its metabolites, or biological parameters, which can suffer alterations by interaction with other biomolecules or compartments of the organism. In this study, the most significant biomarkers derived from exposure to ZEN in both humans and animals or cell line cultures have been included (Figure 1).

**Figure 2:**
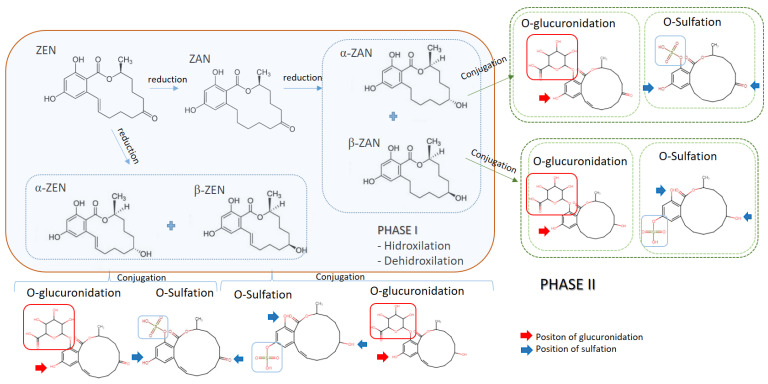
The main active metabolites of ZEN in humans and animals.

ZEN’s reduction is mainly mediated by liver enzymes, but also by the intestinal mucosa or the microbiota, producing zearalanone (ZAN), α-zearalenol (α-ZEL), β-zearalenol (β-ZEL), α-zearalanol (α-ZAL), and β-zearalanol (β-ZAL) [8]. α-ZEL and β-ZEL are the main active metabolites of ZEN in humans and animals, they are generated from the reduction of ZEN. α-ZEL and β-ZEL are considered the major “phase I” metabolites (in addition to the zearalanols α-ZAL and β-ZAL) while “phase II” metabolites are the glucuronide forms (Figure 2). Four glucuronides have been primarily detected: ZEN-14-glucuronide, ZEN-16-glucuronide, ZEN-14,16-diglucuronide, and ZEN-14-2-glucuronide.

Regarding their strogenic activities, α-ZEL is 92 times more potent than the β-ZEL form, while β-ZEL is 2.5 times less potent than the free ZEN molecule [12]. Both metabolites are hydroxylated by 3α or 3β-hydroxysteroid dehydrogenase (HSD) and conjugated with glucuronic acid by uridine diphosphate-glucuronosyltransferase (UDPGT), forming the compounds α and β-zearalenol-14-glucuronide (α and β-ZEL14GlcA). However, ZEN is also transformed into zearalenone-14-glucuronide (ZEN14GlcA) by the enzyme UDPGT, while zearalenone-14-sulphate (ZEN-14-S) is a bioconjugated form of ZEN, which was detected in urine samples of pigs. ZEN and its metabolites detected in biological human samples have been ZEN, α-ZEL, β-ZEL, α-ZAL, β-ZAL, ZEN14GlcA [8,12].

Furthermore, ZEN can be found in food as one of its “modified forms” (covalently or non-covalently bound to the components of the food matrix) and subsequently be digested in the gastrointestinal tract and released as the bioavailable parental mycotoxin [13]. These modified forms produced by plants and fungi comprise of ZEN’s glucoside or sulphated conjugates that can be rapidly reverted to their aglycone forms through the hydrolysis in the gastrointestinal tract [4]. Recently, in an in silico study it was revealed that products describing a metabolomics profile were: O-glucuronidation (1z and 2z for ZEA and 1 ab, 2 ab and 3 ab for ZEA’s metabolites), S-sulphation (3z and 4z for ZEA, and 4 ab, 5 ab and 6 ab for ZEA’s metabolites), and hydrolysis (5z and 7 ab for ZEA’s metabolites, respectively) [14].

Each modified form of ZEN was assigned a relative potency factor (RPF), derived from results of the uterotrophic assay in rats, to be applied to exposure estimates of the respective modified forms to reflect the different in vivo estrogenic potencies. ZEN as reference or index mycotoxin was determined to have a RPF of 1.0, whereas 60 was assigned to α-ZEL in due to its higher estrogenic potency [15]. The major reactions for ZEN in human and animal liver microsomes include reduction, hydroxylation, and glucuronidation, among others. ZEN is rapidly absorbed after oral ingestion and is metabolized by 3α- or 3β-hydroxysteroid dehydrogenase to its α-ZEL and β-ZEL stereoisomers, the major phase I metabolites. ZEN, α-ZEL, and β-ZEL are metabolized by uridine diphosphate-glucuronosyltransferase (UDPGT) and sulphotransferase (SULT) in the liver and/or intestinal cells to give rise to phase—metabolites that will be excreted by the urine. The reduction of α-ZEL and β-ZEL occurs mainly in the liver but it has been shown that erythrocytes are also capable of metabolizing ZEN as well as the intestinal mucosa or microbiota [16].

With regard to the porcine test, ZEN elimination was shown, which mainly occurs in feces (40%) compared to urine (26%), on its free form and followed by metabolites ZEN-14 -GlcA, and α-ZEL [17].

The main objective of this review is to gather the latest literature information about ZEN biomarkers in humans, animals, or cell line cultures but also to describe which biomarkers permit to more reliably determine the magnitude of exposure to ZEN through diet. Then, it will provide information of interest to understand the interpretation of biomarker levels and their relationship with the amount of ZEN ingested by the population.

## 2. Biomarkers of ZEN in Urine

ZEN and its metabolites are excreted in urine mainly as conjugated forms with glucuronic acid or sulphates. These metabolites help to estimate the exposure to ZEN with urine and using them as biomarkers. Among that, when urinary samples are processed, it is necessary to perform enzymatic hydrolysis, specifically, β-glucuronidase and sulphatase. Such enzymatic hydrolysis helps to not underestimated urinary levels [18]. The intention of these studies is to establish the provisional maximum tolerable daily intake (PMTDI) of the population and the inhabitants of different demographic areas. In most cases, studies of ZEN intakes, the levels of ZEN, and its main metabolites have been reported below the TDI of 0.25 μg/kg bw for total ZEN [5].

A recent study of urine samples in men and women of the Rajshahi city (Bangladesh) (urban and rural population) and in pregnant women from the Dhaka (Bangladesh, two seasons) has been carried out [19]. Urinary parameters of these samples were collected. Regarding creatinine levels (CRE) values, no statistically significant differences were observed between rural/urban men and women, except when comparing summer and winter seasons on which a high fluid intake in warm temperatures was observed; while in the group of pregnant women, there were no significant differences in CRE levels. Regarding ZEN metabolites, α-ZEL turned out to be the metabolite highly present in urinary samples from adults (compared to the parental mycotoxin, ZEN) compared to β-ZEL, which was in a much lower quantity. This fact placed α-ZEL as an important biomarker of exposure to ZEN. In urine samples from pregnant women, β-ZEL was detected at concentrations higher than in the other cohorts. Among them, the concentrations of the biomarkers of exposure to ZEN were higher in the winter season than in the summer season for both rural and urban populations. Values of the total content of ZEN (added to that of its metabolites) were less than 0.25 ng/mL for almost all samples. Winter urine samples of rural adults in Bangladesh in winter yielded the highest content of biomarkers of exposure to ZEN; while in pregnant women, the presence of β-ZEL in all samples was highlighted. It was reported that variations in the levels of metabolites in the urine of pregnant women were associated with the alteration of endogenous steroid hormones. It is based on the fact that these hormones affect the activity of hydroxysteroid dehydrogenase, responsible for the conversion of ZEN into its phase I metabolites, and the changes in the eating habits adopted during pregnancy [19].

The different ranges of urinary biomarkers from various countries show that the exposure to mycotoxins depends on many factors that affect the dietetic habits, with the geographical region and age as the most influential. In African countries, maize is a staple food and the levels of ZEN in urine were higher than those in European countries where the estimated exposure to ZEN is much lower and the dietary pattern includes wheat and maize. However, in Bangladesh (Asia) the staple food is rice and ZEN and its metabolites in urine were lower than in European countries [19]. Furthermore, the age is decisive, as in the case of children where a higher intake of ZEN in proportion to the individual’s body weight was observed, which lead to concentrations higher than those obtained in urine from adults from the same geographical area.

Two complementary studies have been published by Gambacorta et al. [20] and Solfrizzo et al. [13]. One evaluated the exposure biomarkers of ZEN in pigs [20] and the other established the probable dietary intake (PDI) in an Italian region [13]. In the first, the dose-response relationship that existed between oral ingestion and the urinary excretion of their most relevant biomarkers in the next 24 h was evaluated. It should be noted that the α-ZEL/β-ZEL ratio attained a value of 36.5, showing that the first metabolite was much more abundant in urine samples than the second one. In addition, it was possible to calculate the percentage of ingested mycotoxin that was excreted as biomarkers in the urine of 24 h after a dose, and in the case of ZEN, it was 36.8%. These results served Solfrizzo et al. [13] to calculate the PDI of ZEN based on the concentration of the urinary biomarker in samples from 52 volunteers from the Apulia region of southern Italy. ZEN and α-ZEL were detected in 100% of the urine samples while β-ZEL was present in 98% of the samples. Biomarker concentration means for ZEN, α-ZEL, and β-ZEL were 0.057, 0.077, and 0.090 ng/mL, respectively. The low concentrations of ZEN in all samples were related to the low dietary intake of ZEN in the Italian population.

A great application of this urinary mycotoxin’s biomarker studies is the estimation of the PDI. This estimation is based on the following equation:

$$\mathrm{PDI}=C\;\times \frac{V}{W}\times \frac{100}{E}$$

C: Concentration of the biomarker in human urine (µg/L)

V: 24-h mean urinary volume (1.5 L)

W: Average human weight (60 kg)

E: Average mycotoxin excretion ratio in 24 h post-intake in pigs (36.8% ZEN).

The main difference between EDI and PDI is that the latter is based on the measurements of biomarkers concentrations and represents the value to provide an approximate estimation level of consumption. It is important to note that PDI is often compared with TDI to estimate the potential risk of exposure of the consumer to the mycotoxins. In particular, if PDI surpassed TDI, further enquiries must be made in order to identify the source of contamination [8].

Until now, the estimation of the PDI for ZEN has been calculated using data of swine excretion by Gambacorta et al. [20] evidencing the lack of representative and large-scale studies on the pattern of excretion of ZEN in humans [18].

Recently, the urinary excretion of ZEN has been monitored in a male volunteer exposed to a daily dose of 10 μg of ZEN (represents about 2/3 of the TDI of 0.25 μg/kg bw/day) as part of a naturally contaminated diet on four consecutive days [4]. During the exposure period, the concentrations of ZEN in urine were found in the range of 0.30–0.59 ng/mL (mean 0.39 ng/mL), which, multiplied by the daily urinary volume, meant a mean excretion ratio of 9.4%. Therefore, the urinary excretion of ZEN in humans is approximately 10% of the administered dose, while in the case of pigs it is around 36.8% [21]. However, to make a correct approximation when converting urinary concentrations of ZEN biomarkers into provisional data of PDI in humans, it will be necessary to obtain better data on their kinetics in humans.

These types of studies show the levels of diversity in geographic areas, as in a study in different areas of New Jersey (USA) in which a high range of biomarker concentrations was observed in urine [22]. Values were between 4–32 times higher (1.82 ng/mL), one value higher than those obtained by Solfrizzo et al. [13]. There are also results from the German population (PDI was 0.107 μg/kg bw/day), which are higher than those obtained from the Italian population and lower than the levels determined in African countries. The lowest levels of biomarkers were observed in the Swedish population [4].

All these studies reveal not only the excretion of ZEN in urine after controlled exposure but also the possibility of following its metabolization, the diversity among countries and groups of the population, and at the same time the lack of broader studies.

## 3. Biomarkers of Kidney Effects by ZEN

The monitoring in the blood of urea nitrogen (BUN), uric acid (UA), or creatinine (CRE) can be of great interest to determine the renal toxicity of exposure to ZEN, due to its high predictive capacity associated with tissue necrosis, protein catabolism and kidney function. Changes in BUN levels have been described in most cases of nephrotoxicity, including those that are produced by mycotoxins. An increase in BUN levels represents a decrease in the glomerular filtration rate (GFR) and an increase in nitrogen metabolism is associated with decreased renal blood flow. On the other hand, CRE is an analytical parameter used to estimate glomerular filtration, as well as the presence of kidney dysfunction or muscle disorder. These parameters are considered adequate in reflecting the degree of kidney damage [23].

Jia et al. [23] studied the effect of four levels of ZEN on pregnant rats that received a daily intake of 0.3, 48.5, 97.6, and 146.0 mg/kg, respectively (equivalent to 0, 4.5, 9, and 13.5 mg/kg bw/day). Animals were followed and during the first seven days of gestation, it was observed how concentrations of BUN, CRE, and UA were modified in serum and urine (Table 1). Exposure to ZEN significantly affected some biomarkers of kidney function.

The intensity of these changes was dependent on the dose of ZEN administered as follows: (i) the plasma BUN and UA levels were significantly increased in the groups that intake 97.6, and 146.0 mg/kg; (ii) the plasma/urine CRE level decreased in all the ZEN treated groups; and (iii) the level of UA in urine was increased in all groups treated with ZEN [23].

Other researchers such as Liang et al. [24] evaluated the levels of CRE and BUN in nine groups of mice, concluding that the serum levels of CRE and BUN were significantly increased in all groups treated with DON or ZEN (or both simultaneously) three to five days after treatment compared to the control group. In particular, it should be noted that the serum levels of CRE and BUN were maximum in the group treated with 2.5 mg/kg DON + 30 mg/kg ZEN. Therefore, it revealed a nephrotoxic additive effect for the mixture of these two mycotoxins. The same article indicates that the discrepancies observed for CRE levels in other studies, such as the one already referenced by Jia et al. [23], can be attributed to the differences in animal species used in the experiment, the route of exposure, the ingestion dose or the exposure period. This can help to indicate that all three parameters (BUN, UA, and CRE) are good parameters for evaluating and comparing kidney effects by ZEN.

**Table 1:** In vivo studies of ZEN´s biomarkers.

Mycotoxin	Study Population	Sample Analyzed	Parameters Measured	Exposed Concentration/Time	Result and Interpretation	Reference
ZEN	20 post-weaning piglets (Landrace × Yorkshire × Duroc)	Blood Isolated organs	-Body weight -Vulva size -Conc. AST, ALT, ALP, GGT, Urea, CRE -Oxidative stress: SOD, GSHPx, MDA	Fed a basal diet ZEN1: 1 mg/kg ZEN2: 2 mg/kg ZEN3: 3 mg/kg 18 days	-Invariable body weight -↑ Vulva size by 277% (ZEN1)/643% (ZEN2)/762% (ZEN3) -↑ Conc. Aminotransferases and ALP + Urea + CRE -↓ SOD/GSHPx enzyme activity and ↑ MDA àHyperestrogenism, liver damage and induction of renal tissue degeneration, stimulation of oxidative stress and lipid peroxidation	[25]
ZEN	9 week old Sprague-Dawley rats Pregnants Rats	Blood Urine Kidney	-BUN, CRE and UA levels in blood/urine -Oxidative stress: MDA, SOD, GSPHx -Renal histopathological examination -Total RNA and PCR quantification	Fed a basal diet ZEN at 50/100/150 mg/kg On gestation days 0 through 7	-↑ BUN/UA levels in plasma and ↓ CRE levels in plasma/urine -↑ MDA and ↓ SOD/GSHPx activity -Protein deposits, interstitial fibrosis, tubular degeneration, shrunken glomerulus -↑ expression of pro-inflammatory cytokines TLR4, NF-kBp65, TNF-α, IL-1β, IL-6, and β-actin	[23]
ZEN	Male Wistar Crl:WI BR rats (8 weeks of age)	Kidney and Liver	-Body weight -Absolute and relative weight of liver and kidney -Lipid composition of the liver/kidney membrane -Hepatic and kidney antioxidant parameters	Fed Ssniff R/M-Z+H feed FB1: 150 µg/animal/day; DON: 30 µg/animal/day; ZEN: 15 µg/animal/day Toxins binary (FD, FZ, DZ) and ternary (FDZ) 14 days	-Initial = final weight -The group fed ZEN experienced ↑ absolute kidney size -Renal lipid profile: ↑ á. linoleic, ↑ á.gamma-linoleic and ↑ á. gondoic	[1]
ZEN	Wistar rats (10 wk of age)	Blood	-Hepatotoxicity (Aminotransferases) -Hematotoxicity	ZEN: 1.5, 3, 5 mg/kg/day 28 days	-↑ Conc. Aminotransferases and ALP -Alteration of hematological parameters	[26]
ZEN	Female Kunming mice	Blood	-Blood CRE and BUN levels -Oxidative stress: SOD and hydroxyl radical inhibitory capacity -MDA and NO -Renal apoptosis	Intraperitoneal injection ZEN: Z1 (20 mg/kg body weight), Z2 (30mg/kg body weight), DON: D1 (1.5 mg/kg body weight), D2 (2.5 mg/kg body weight) The binary mixtures D1Z1/D1Z2/D2Z1/D2Z2 12 days	-↑ CRE and BUN levels -↓ SOD enzyme activity and -OH inhibitory capacity -↑ MDA and NO in all groups treated with ZEN and DON -↑ rate of renal apoptosis in all treated groups except those that received low doses of ZEN	[24]
ZEN, α-ZEL and β-ZEL	52 volunteers, Apulia region (Italy) Adults	Urine	-Levels of ZEN and its main metabolites (α-ZEL and β-ZEL)	PDI aprox for ZEN: 0.015 µg/kg bw/day	-Mean of urine levels: ZEN: 0.057 ng/mL; α-ZEL: 0.077 ng/mL; β-ZEL: 0.090 ng/mL -Provisional PDI < TDI (0.2 µg/kg bw/day)	[13]
ZEN, α-ZEL and β-ZEL	60 volunteers from Dortmund (Germany) Adults	Urine	-Levels of ZEN and its main metabolites (α-ZEL and β-ZEL)	2 weeks 6 weeks 7 weeks	-Means of urine levels: ZEN 0.10 ± 0.05 ng/mL; α-ZEL 0.16 ± 0.07ng/mL; β-ZEL 0.05 ± 0.04 ng/mL -Comparison with other urinary biomarker studies	[4]
ZEN, α-ZEL and β-ZEL	Rural area (Mongol Para) and urban area (Rajshahi) in the Rajshahi district of Bangladesh. Adults	Urine	-Levels of ZEN and its main metabolites (α-ZEL and β-ZEL)	2 periods: winter and summer	-Mean of urine levels: α-ZEL (0.338 ± 0.252 ng/mg CRE) >> ZEN (0.064 ± 0.060 ng/mg CRE) >> β-ZEL (0.029 ± 0.025 ng/mg CRE) -Pregnant: ZEN: 0.185 ± 0.187 ng/mg CRE; α-ZEL: 0.516 ± 0.484 ng/mg CRE; β-ZEL: 0.148 ± 0.146 ng/mg CRE	[19]

## 4. Biomarkers of Liver Effects by ZEN

ZEN is metabolized in the first instance in the liver, so that of the most common toxic effects produced by this mycotoxin is liver damage. Considering a study carried out by Jiang et al. [25], it is worth highlighting the significant increase in serum levels of alanine aminotransferase (ALT), aspartate aminotransferase (AST) and gamma-glutamyltransferase (GGT), and alkaline phosphatase (ALP) in animals treated with ZEN supplemented diets. The animals studied were 20 piglets that were fed with 1, 2 and 3 mg/kg of ZEN. In addition, an increase in the size of the liver and lung was observed, being the splenic size the only exception (reduced with the administration of ZEN).

Other studies supported the hepatotoxicity of ZEN through aminotransferase values that were multiplied by up to 100 times compared to the animals that received the control treatment [26]. In this case, female rats received an intraperitoneal injection of ZEN at a dose of 1.5, 3, and 5 mg/kg, and after 48 h, a blood test was performed. As described, the values of the main aminotransferases (AST/ALT) and ALP were greatly altered, demonstrating the hepatotoxic power of this mycotoxin in this organ.

## 5. Cellular Response by ZEN Exposure

The response of ZEN at the cellular level has been reported by several studies and this section is collecting the effects on oxidative stress, cell heat shock protein (Hsp), cell membrane effects, and gene expression.

### 5.1. Biomarkers of Oxidative Stress

Oxidative stress is the answer to the disbalance between the production of reactive oxygen species (ROS) and a biological system’s ability to detoxify or repair the resulting damage. The ROS can cause lipid peroxidation, degradation of cytosolic protein, and damage to DNA, leading to cell death as an ultimate consequence. More recently, there is a wide number of studies evidencing the effects of oxidative stress damage caused by ZEN on different cell lines [27,28,29,30]. The ROS hydrogen peroxide (H_2_O_2_) is decomposed along with glutathione peroxidase (GPx) and catalase (CAT) enzymes. CATs are abundant in the peroxisomes of liver cells, and GPx is abundant in mitochondria and cytosol compartments. Agahi et al. [30] evaluated the CAT activity in undifferentiated SH-SY5Y cells treated with α-ZEL and β-ZEL (1.56, 3.12, 6.25, and 12.5 µM) and a significant decrease in all individual treatments at 24 h, as well as in combinations, was observed.

ZEN is capable of generating oxidative free radicals in a dose-dependent manner and also altering the expression of antioxidant enzymes and genes related to oxidative stress both in vivo and in vitro. In fact, it was observed that exposure to ZEN induces the gene expression of O-6-Methylguanine-DNA Methyltransferase (MGMT), α- glutathione S-transferase (GST), heat shock protein 70 (Hsp70), and heme oxygenase-1 (HO-1) in human cell lines [31].

The production of ROS was studied in some trials; in one carried out by Lei et al. [3], PK-15 cells were exposed for 24 h to certain concentrations of ZEN (10 μM), among other mycotoxins: aflatoxin B_1_ (AFB_1_), deoxynivalenol (DON), and fumonisin B_1_ (FB_1_), observing synergistic and antagonistic effects in the administration of some binary mixtures. In the binary mixture study, with AFB_1_ and ZEN, they showed that ZEN enhanced the ROS production caused by 1 µM AFB_1_ and displayed synergistic effects in combination with AFB_1_ at 5 and 10 µM. Low levels of AFB_1_ were antagonistic to ZEN, but high doses of AFB_1_ displayed synergistic effects on oxidative damage with ZEN [3].

Regarding enzyme activity, decreased activity of the enzymes superoxide dismutase (SOD) and glutathione peroxidase (GPx) in both serum and liver suggests induction of oxidative stress in the liver and other physiological tissues. The associated increase in malondialdehyde (MDA) as a metabolite of the lipid peroxidation process is directly associated with the reduction of the enzymatic antioxidant activity of SOD/GPx in response to dietary supplementation (1, 2, or 3 mg/kg of ZEN for 18 d ad libitum) in the diet fed with ZEN [25].

In 2014, Jia et al. [23] focused on determining some oxidative parameters at the plasma and renal level in response to exposure to ZEN. Specifically, MDA concentrations, as well as the enzymatic activities of SOD and GPx, were assessed both in the kidney and in the plasma of mice fed with 0.3, 48.5, 97.6, 146.0 mg/kg of ZEN. As a conclusion, both ZEN 100 (97.6 mg/kg) and ZEN 150 (146.0 mg/kg) groups showed reduced activities of the enzymes SOD and GPx and increased concentrations of MDA in the plasma (*p* < 0.05). Afterwards, it was observed in these animals how oxidative stress is promoted in response to exposure to ZEN in the same animals.

These studies show how ZEN breaks the pro-oxidant and antioxidant balance to end up producing oxidative damage. Numerous articles described the study of altered antioxidant parameters after exposure to mycotoxins. Concretely, Liang et al. [24] measured the enzymatic activity of SOD as well as the inhibitory capacity of hydroxyl radicals (-OH). It was observed that the activities of SOD and the hydroxyl radical inhibitory capacity decreased significantly in all the treated groups compared to the control after three to five days of treatment and, from that moment, gradually increased after eight days from treatment. In addition, this fact was associated with the increase in other compounds derived from oxidative stress such as MDA or nitric oxide (NO), which increased their serum levels in all groups treated with ZEN and DON exposed for long periods and in a dose-dependent manner. It can be concluded that DON and ZEN are disruptors of the pro-oxidant and antioxidant balance.

The oxidative stress caused by exposure to the two major metabolites of ZEN, α-ZEL and β-ZEL was also studied in the in vitro field. The ability of the aforementioned metabolites to induce lipid peroxidation in a dose-dependent manner was verified [16]. To quantify this fact, the levels of the MDA induction factor in Vero cells were measured and these were multiplied by five and even seven times concerning the levels of the control group.

The exposure impact of human cells to certain doses of ZEN has also been studied. Karaman et al. [31] tried to elucidate the effects of ZEN in a human renal tubular proximal cell line (HK-2 cells) after 24 h of exposure. Measuring the intensity of the fluorescence generated, it was seen how ZEN, at concentrations from 1.10 to 50 µM, was able to significantly induce the generation of oxidative free radicals in ratios of 27.69, 64.24, and 103.82%, respectively, compared to the control group analyzed in HK-2 cells after 24 h of exposure.

### 5.2. Heat Shock Protein

Heat shock proteins (Hsp), such as Hsp27 and Hsp70, are considered the first line of action in the cellular response to exposure to toxic substances. Therefore, it is appropriate to use it as an easily measurable marker of toxicity since its expression is increased in the face of harmful events that require increased cytoprotection of the organism (Table 2).

The results obtained by Bouaziz et al. [32] showed that ZEN in combination with mycotoxin T-2 was capable of inducing a concentration-dependent increase in the levels of Hsp70. This study shows the importance of cellular and/or oxidative damage that the binary mixture of this type of mycotoxins is capable of producing.

The strong correlation between oxidative stress and the expression of Hsp has been demonstrated by other authors. Therefore, authors such as Othmen et al. [16] provided relevant data on the dose-dependent increase in the production of Hsp27 and Hsp70 in Vero cell cultures exposed to α-ZEL/β-ZEL, concentrations that induced 10.20 and 50% losses in cell viability. Furthermore, β-ZEL showed higher induction of these stress proteins compared to α-ZEL.

In the study by Karaman et al. [31], Hsp70 was also measured. From this, the conclusion was drawn that an exposure of 24 h at ZEN was capable of inducing a dose-dependent increase (1, 10 and 50 µM) of the concentrations of this biomarker.

### 5.3. Cellular Membrane Effects

Effects of ZEN in cell membranes have been studied in two interrelated directions: the membrane integrity by lactate dehydrogenase (LDH) measurement assay and the lipid profile composition.

The integrity of the cell membrane can be assessed by quantifying the amount of the cytosolic enzyme lactate dehydrogenase (LDH) that has been released into the environment after exposure to a certain exogenous substance. In this sense, in a study by Lei et al. [3], it was determined that with a ZEN concentration of 84.2 µM, it was possible to increase the release of LDH up to 130% (the study was performed with PK-15 porcine kidney cells incubated with sterile solutions of isolated mycotoxins).

Another factor that has recently been investigated is the lipid profile of the cell membranes of various tissues in the body. An in vivo study published by Szabó et al. [1] reported the effects of purified mycotoxins (ZEN, DON, FB_1_, and their binary and tertiary mixtures), constructing a multitoxic study (14 days of exposure duration) to determine toxic effects and interactions in target organs such as the liver or kidney. In this study, modifications were observed in the lipid composition of the renal tissue membrane, the proportions of some fatty acids were increased: the group treated with ZEN presented an increase in linoleic acid (C18:2 n-6), gamma-linoleic acid (C18:3 n-6), and gondoic acid (C20:1 n-9).

### 5.4. Effect in Gene Expression

ZEN has also been attributed the ability to induce genotoxicity in experimental animals or some cell line cultures. Jia et al. [23] measured the expression levels of toll-like receptor 4 (TLR4), nuclear factor kBp65 (NF-kBp65), tumor necrosis factor (TNF-α), the interleukines IL-1β and IL-6. Compared to the control, there was a significant increase in the mRNA expression of TLR4 and NF-kBp65 in all treated groups. Similarly, there was an increase in mRNA levels of inflammatory cytokines (TNF-α, IL-1β, and IL-6) in groups ZEN treated at 97.6 and 146.0 mg/kg, respectively, in a dose-dependent manner.

As mentioned above, oxidative stress is a mechanism of toxicity caused by ZEN. Karaman et al. [31] studied the analysis of the expression of certain genes related to oxidative stress in HK-2 cells such as MGMT, α-GST, Hsp70, nuclear factor Nrf2, fatty acid-binding protein L-Fabp, HO-1, and of certain inflammatory cytokines such as IL-6, IL-8, TNFα, NFkB, and MAPK8. After exposure of ZEN at different concentrations for 24 h, an increase in genes expression was manifested, these genes were: MGMT, α-GST, Hsp70, and HO-1. However, the L-Fabp expression was reduced concerning the expression levels of inflammatory cytokines, and an increase in IL-6, IL-8, TNFα, and MAPK8 was found [31].

Then, ZEN exposure produces kidney damage and the previous biomarkers have a specific effect on interpretation, the presence of which is interesting. α-GST is usually determined in proximal tubular cells when its levels rise in urine and it has demonstrated its diagnostic utility as a liver injury biomarker; on the other hand, HO-1 is responsible for the degradation of the heme group and is regulated by Nrf2 in the proximal tubule, their alteration after ZEN exposure has been observed [31]. They exert a cytoprotective and nephroprotective role in response to kidney damage. MGMT, one of the key enzymes in DNA repair, is related to oxidative stress and its increase allows the formation of reactive oxygen species. For all this, the aforementioned genes can be used as renal biomarkers in vitro.

L-Fabp is responsible for protecting the kidney from oxidative tubular damage but, as its levels are reduced due to ZEN exposure, the amount of mRNA responsible for its encoding is scarce and leads to kidney damage [31].

MicroRNAs constitute a specific class of small (approximately 22 nucleotides long) non-coding RNAs. They regulate gene expression at the post-transcriptional level by mRNA cleavage or translational repression [33]. Due to the release of microRNAs from tissue to extracellular biofluids, microRNAs have remarkable potential for biomarker development. Measurement of circulating microRNAs, e.g., in serum, urine, or saliva, can serve as an indicator for pathological processes in tissues. Thus, microRNAs represent a new generation of predictive biomarkers for toxin exposure and disease [34,35]. In contrast, research on the impact of endocrine disruptors on microRNA expression is still in its early stages [36,37]. For ZEN, only two reports are available, both focusing on pigs and employing targeted approaches for microRNA quantification (qPCR on a few selected microRNAs): Brzuzan et al. [38] and He et al. [39]. Recently, the increased usage of ‘omics´ technologies was proposed for addressing existing mechanistic knowledge gaps in endocrine disruptor research [40]. Grenier et al. [41] studied piglets that received contaminated feed over 28 days, and 14 microRNAs were commonly and dose-dependently affected in both the ZEN medium (1.46 mg/kg) and ZEN high (4.58 mg/kg) group, including microRNAs from the miR-503 cluster (e.g., ssc-miR-424-5p, ssc-miR-450a, ssc-miR-450b-5p, ssc-miR450c-5p, ssc-miR-503, and ssc-miR-542-3p). Predicted target genes for those microRNAs are associated with regulation of gene expression and signal transduction (e.g., cell cycle). Although the effects in serum were less pronounced, receiver operating characteristic analysis revealed that several microRNA ratios were able to discriminate properly between non-exposed and ZEN-exposed pigs (e.g., ssc-miR135a-5p/ssc-miR-432-5p, ssc-miR-542-3p/ssc-miR-493-3p).

## 6. Biomarkers of Reproductive Endocrine Effects

One of the major toxic effects of ZEN in the human body and/or animals is a reproductive and endocrine disruptor since it acts as an estrogenic analogue, due to its affinity with estrogenic receptors (ERs).

After dimerizing with the ER, the ZEN enters the nucleus and begins the transcription of genes and the synthesis of related proteins, causing endocrine disruption that leads to toxicity [1].

Several studies mention the direct effects of ZEN in the reproductive organs of different animal species. This has been revealed in a study with piglets, where an increase in the size of the vulva of 277%, 643%, and 762% were observed for ZEN purified at 1, 2 and 3 mg/kg fed a basal diet [25]. An analysis of the contents of ZEN and its main derivatives on a population of wild female warthogs in Poland [42] confirmed the effect that these may have on the reproductive system. In particular, this study was carried out to identify the causality of reproductive disorders and sexual dysfunctions that had been observed in wild boars in this geographical area. To do this, the concentrations of ZEN and its derivatives were determined in organs, tissues, and body fluids of these young females distributed in the two major habitats farmland or forests. Females aged one–two years were used since in previous studies with porcine species it had been confirmed as the age of greatest sensitivity to the estrogenic and/or hormonal effects caused by ZEN. The highest concentrations of the compounds analyzed were obtained in urine and bile, demonstrating the rapid metabolism of ZEN into elimination compounds rapidly excreted from the body. In turn, the initial hypothesis was confirmed, since the wild boars that inhabited the farmlands and fed in large cornfields were those that presented a greater accumulation of the estrogenic mycotoxin ZEN.

The hormonal alterations that these animals suffered were: infertility, reproductive cycle disturbances, delay/prolongation of ovulation, and in some cases hyperestrogenism induced by excessive estrogenic stimulation. In addition, morphological changes such as uterine and mammary glands enlargement were also reported. On the other hand, alterations such as vaginal/straight prolapse, abortions and low pregnancy rates, among others, have been reported in porcine species. For ZEN contents in feed greater than 1 mg/kg, a decrease in the integrity of the follicles and a generalized increase in the expression of estrogenic receptors has been evidenced in the case of lactating pigs [43].

## 7. Biomarkers of Hematologic Effect

ZEN has also been classified as a mycotoxin capable of producing haematotoxic effects. According to Maaroufi et al. [26], after an intraperitoneal administration of doses of 1.5, 3, and 5 mg/kg of ZEN in female rats and the subsequent analysis of their blood samples after 48 h, a blood coagulation dysfunction was observed in these rats and also some blood parameters were altered (hematocrit, mean corpuscular volume, number of platelets or white blood cell count). The hematocrit of the rats showed significantly increased values compared to the control group and, in turn, with differences between each of the groups treated with different doses of ZEN. It was seen that the mean corpuscular volume (MCV) gradually increased as the administered dose of ZEN was higher, while the red blood cell count (RBC) remained constant. The hemoglobin content was slightly higher in the groups treated with 3 and 5 mg/kg of ZEN and the platelet count showed a very significant decrease in each of the treated groups, indicating a possible influence of the ZEN on blood clotting [26]. Finally, the white blood cell count reached higher values at least in the groups treated with the highest doses (3 and 5 mg/kg).

## 8. Conclusions

α-ZEL and β-ZEL, in addition to free ZEN, are the most commonly used urinary biomarkers to identify exposure to ZEN through food. Using ZEN as a urinary biomarker, it is possible to estimate the probable dietary intake (PDI). However, more studies are needed to define the excretion rate of ingested ZEN as urinary biomarkers by human age groups.

The biomarkers that reflect the cellular damage caused by ZEN or their metabolites α-ZEL and β-ZEL on the different studied cells and tissues of the organism both in vivo and in vitro are: (i) oxidative stress, (ii) cell membrane (LDH activity and lipid profile); (iii) inhibition of macromolecule synthesis. It is concluded that α-ZEL and β-ZEL are cytotoxic by inhibiting cell proliferation and total protein and DNA syntheses. In addition, ZEN metabolites induce Hsp27 and Hsp70; gene expression (TLR4, NF-kBp65, TNF-α, IL-1β, IL-6, IL-8, MGMT, α-GST, Hsp70, Nrf2, L-Fabp, HO-1, MAPK8); and (iv) hematotoxicity (presenting variations in hematocrit, mean corpuscular volume, hemoglobin, platelets, and white blood cells).

The biomarkers that best reflect the organ damage caused by ZEN are those related to oxidative stress (catalytic activity of SOD and GPx, levels of MDA and NO), nephrotoxicity (CRE, BUN, and UA levels, and histopathological changes in the kidney), hepatotoxicity (levels of hepatic aminotransferases AST, ALT, GGT, and alkaline phosphatase ALP), and reproductive toxicity (size of female genital organs). It has been noticed that the relative weight of genital organs, liver, and kidney increased linearly in a ZEA-dose-dependent manner.

This review includes those studies that correlate effect and exposure to ZEN through biomarkers, which will allow starting studies of animal and population exposure in parallel with those of effect works.

## Figures and Tables

**Table 2:** In vitro studies of ZEN´s biomarkers.

Mycotoxin	Sample Analyzed	Evaluated Effect	Exposed Concentration/Time	Result and Interpretation	Reference
ZEN	PK-15 cells	-LDH release to the medium (membrane integrity marker) -Oxidative stress: ROs production -Induction of apoptosis	ZEN: 71–99 µM AFB1: 20–34 µM DON: 0.6–3.4 µM Tertiary combination. At 8 a.m., 12 h, 24 h and 48 h	-84.2 µM ZEN ↑ in 130% LDH release -ROs: ZEN/↓ AFB1 antagonism and ZEN/↑ AFB1 synergism -Additional effect of 40 µM ZEN + 1 µM AFB1 in late apoptosis	[3]
ZEN	Vero cells	-Heat shock protein (HSP 70)	ZEN + T-2 toxin at equimolar concentration of 10 nM and between 0–100 nM	-↑ HSP 70 compared to control	[32]
α-ZEL and β-ZEL	Vero cells	-Macromolecule synthesis -Oxidative stress -Heat shock protein (HSP 27 and HSP 70)	ZEN: 0–150 µM	-↓ protein synthesis and ↓ DNA synthesis -↑ MDA (↑ lipid peroxidation) -↑ HSP27 and ↑ HSP70	[16]
ZEN	HK-2 cells	-Oxidative stress -Gene expression -Heat shock protein (HSP70)	ZEN: 1–10–50 µM ZEN: 50–200 µM	-↑ oxidative stress -↑ expression of genes related to oxidative stress -↑ HSP70	[31]

## Data Availability

The data presented in this study are available in the article.

## References

[1] Szabó A., Szabó-Fodor J., Fébel H., Mézes M., Balogh K., Bázár G., Kocsó D., Ali O., Kovács M. (2018). Individual and combined effects of fumonisin B1, deoxynivalenol and zearalenone on the hepatic and renal membrane lipid integrity of rats. Toxins.

[2] Lee H.J., Ryu D. (2017). Worldwide occurrence of mycotoxins in cereals and cereal-derived food products: Public health perspectives of their co-occurrence. J. Agric. Food Chem..

[3] Lei M., Zhang N., Qi D. (2013). In vitro investigation of individual and combined cytotoxic effects of aflatoxin B1 and other selected mycotoxins on the cell line porcine kidney. Exp. Toxicol. Pathol..

[4] Ali N., Degen G.H. (2018). Urinary biomarkers of exposure to the mycoestrogen zearalenone and its modified forms in German adults. Arch. Toxicol..

[5] EFSA Panel on Contaminants in the Food Chain (2011). Scientific Opinion on the risks for public health related to the presence of zearalenone in food. EFSA J..

[6] Marin S., Ramos A.J., Cano-Sancho G., Sanchis V. (2013). Mycotoxins: Occurrence, toxicology, and exposure assessment. Food Chem. Toxicol..

[7] EFSA (2016). Appropriateness to set a group health-based guidance value for zearalenone and its modified forms. EFSA J..

[8] Al-Jaal B.A., Jaganjac M., Barcaru A., Horvatovich P., Latiff A. (2019). Aflatoxin, fumonisin, ochratoxin, zearalenone, and deoxynivalenol biomarkers in human biological fluids: A systematic literature review, 2001–2018. Food Chem. Toxicol..

[9] IARC (1993). Some Naturally Occurring Substances: Food Items and Constituents, Heterocyclic Aromatic Amines and Mycotoxins. IARC Monographs on the Evaluation of Carcinogenic Risks to Humans, No. 56.

[10] Lee H.J., Ryu D. (2015). JFS special issue: 75 years of advancing food science, and preparing for the next 75: Advances in mycotoxin research: Public health perspectives. J. Food Sci..

[11] Owen R., Galloway T.S., Hagger J.A., Jones M.B., Depledge M.H. (2008). Biomarkers and environmental risk assessment: Guiding principles from the human health field. Mar. Pollut. Bull..

[12] Steinkellner H., Binaglia M., Dall’Asta C., Gutleb A.C., Metzler M., Oswald I.P., Parent-Massin D., Alexander J. (2019). Combined hazard assessment of mycotoxins and their modified forms applying relative potency factors: Zearalenone and T2/HT2 toxin. Food Chem. Toxicol..

[13] Solfrizzo M., Gambacorta L., Visconti A. (2014). Assessment of multi-mycotoxin exposure in southern Italy by urinary multi-biomarker determination. Toxins.

[14] Agahi F., Juan C., Font G., Ana Juan-García A. (2020). In silico methods for metabolomic and toxicity prediction of zearalenone, α-zearalenone, and β-zearalenone. Food Chem. Toxicol..

[15] Lorenz N., Dänicke S., Edler L., Gottschalk C., Lassek E., Marko D., Rychlik M., Mally A. (2019). A critical evaluation of health risk assessment of modified mycotoxins with a special focus on zearalenone. Mycotoxin Res..

[16] Othmen Z.O., Golli E.el, Abid-Essefi S., Bacha H. (2008). Cytotoxicity effects induced by zearalenone metabolites, α zearalenol and β zearalenol, on cultured Vero cells. Toxicology.

[17] Vidal A., Mengelers M., Yang S., de Saeger S., de Boevre M. (2018). Mycotoxin Biomarkers of Exposure: A Comprehensive Review. Compr. Rev. Food Sci. Food Saf..

[18] Zhang S., Zhou S., Gong Y.Y., Zhao Y., Wu Y. (2020). Human dietary and internal exposure to zearalenone based on a 24-h duplicate diet and following morning urine study. Environ. Int..

[19] Ali N., Degen G.H. (2019). Biomonitoring of zearalenone and its main metabolites in urines of Bangladeshi adults. Food Chem. Toxicol..

[20] Gambacorta S., Solfrizzo M., Visconti A., Powers S., Cossalter A.M., Pinton P., Oswald I.P. (2013). Validation study on urinary biomarkers of exposure for aflatoxin B1, ochratoxin A, fumonisin B1, deoxynivalenol and zearalenone in piglets. World Mycotoxin J..

[21] Tuanny L., Mousavi A., In S.H., Fernandes C.A. (2019). Biomonitoring of mycotoxin exposure using urinary biomarker approaches a review. Toxin Rev..

[22] Bandera E.V., Chandran U., Buckley B., Lin Y., Isukapalli S., Marshall I., King M., Zarbl H. (2011). Urinary mycoestrogens, body size and breast development in New Jersey girls. Sci. Total Environ..

[23] Jia Z., Liu M., Qu Z., Zhang Y., Yin S., Shan A. (2014). Toxic effects of zearalenone on oxidative stress, inflammatory cytokines, biochemical and pathological changes induced by this toxin in the kidney of pregnant rats. Environ. Toxicol. Pharmacol..

[24] Liang Z., Ren Z., Gao S., Chen Y., Yang Y., Yang D., Deng J., Zuo Z., Wang Y., Shen L. (2015). Individual and combined effects of deoxynivalenol and zearalenone on mouse kidney. Environ. Toxicol. Pharmacol..

[25] Jiang S.Z., Yang Z.B., Yang W.R., Gao J., Liu F.X., Broomhead J., Chi F. (2011). Effects of purified zearalenone on growth performance, organ size, serum metabolites, and oxidative stress in postweaning gilts. J. Anim. Sci..

[26] Maaroufi K., Chekir L., Creppy E.E., Ellouz F., Bacha H. (1996). Zearalenone induces modifications of hematological and biochemical parameters in rats. Toxicon.

[27] Tatay E., Espin S., García-Fernandez A.J., Ruiz M.J. (2017). Oxidative damage and disturbance of antioxidant capacity by zearalenone and its metabolites in human cells. Toxicol. Vitro.

[28] Juan-García A., Carbone S., Ben Mahmoud M., Sagratini G., Mañes J. (2020). Beauvericin and ochratoxin A mycotoxins individually and combined in HepG2 cells alter lipid peroxidation, levels of reactive oxygen species, and glutathione. Food Chem. Toxicol..

[29] Taroncher M., Pigni M.C., Diana M.N., Juan-García A., Ruiz M.J. (2020). Does low concentration mycotoxin exposure induce toxicity in HepG2 cells through oxidative stress?. Toxicol. Mech. Methods.

[30] Agahi F., Juan-García A., Font G., Juan C. (2021). Study of enzymatic activity in human neuroblastoma cells SH-SY5Y exposed to zearalenone’s derivates and beauvericin. Food Chem. Toxicol..

[31] Karaman E.F., Ariman I., Ozden S. (2020). Responses of oxidative stress and inflammatory cytokines after zearalenone exposure in human kidney cells. World Mycotoxin J..

[32] Bouaziz C., Bouslimi A., Kadri R., Zaied C., Bacha H., Abid-Essefi S. (2013). The in vitro effects of zearalenone and T-2 toxins on Vero cells. Exp. Toxicol. Pathol..

[33] Bartel D.P. (2004). MicroRNAs: Genomics, biogenesis, mechanism, and function. Cell.

[34] Schraml E., Hackl M., Grillari J. (2017). MicroRNAs, and toxicology: A love marriage microRNAs in liquid biopsies are minimal-invasive biomarkers for tissue-specific toxicity. Toxicol. Rep..

[35] Ghai V., Wang K. (2016). Recent progress toward the use of circulating microRNAs as clinical biomarkers. Arch. Toxicol..

[36] Klinge C.M. (2015). miRNAs regulated by estrogens, tamoxifen, and endocrine disruptors and their downstream gene targets. Mol. Cell. Endocrinol..

[37] Cameron B.E., Craig P.M., Trudeau V.L. (2016). Implication of microRNA deregulation in the response of vertebrates to endocrine-disrupting chemicals. Environ. Toxicol. Chem..

[38] Brzuzan P., Woźny M., Wolińska-Nizioł L., Piasecka A., Florczyk M., Jakimiuk E., Góra M., Łuczyński M.K., Gajecki M. (2015). MicroRNA expression profles in liver and colon of sexually immature gilts after exposure to *Fusarium* mycotoxins. Pol. J. Vet. Sci..

[39] He J., Zhang J., Wang Y., Liu W., Gou K., Liu Z., Cui S. (2018). MiR-7 Mediates the zearalenone signaling pathway regulating FSH synthesis and secretion by targeting FOS in female pigs. Endocrinology.

[40] Messerlian C., Martinez R.M., Hauser R., Baccarelli A.A. (2017). ‘Omics’ and endocrine-disrupting chemicals—new paths forward. Nat. Rev. Endocrinol..

[41] Grenier B., Hackl M., Skalicky S., Thamhesl M., Moll W.D., Berrios R., Schatzmayr G., Nagl V. (2019). MicroRNAs in porcine uterus and serum are affected by zearalenone and represent a new target for mycotoxin biomarker discovery. Sci. Rep..

[42] Pałubicki J., Kosicki R., Twarużek M., Ałtyn I., Grajewski J. (2021). Concentrations of zearalenone and its metabolites in female wild boars from woodlands and farmlands. Toxicon.

[43] Yang C., Song G., Lim W. (2020). Effects of mycotoxin-contaminated feed on farm animals. J. Hazard. Mater..

